# Patient, disease, and survival outcomes for stage IB to stage IV cervical cancer—A population study

**DOI:** 10.1177/17455057231164551

**Published:** 2023-04-13

**Authors:** Christine Wang, Beverly Lester, Longlong Huang, Shaun Sun, Jenny J Ko

**Affiliations:** 1 Department of Surgery, Faculty of Medicine, University of Calgary, Calgary, AB, Canada; 2 Faculty of Medicine, The University of British Columbia, Vancouver, BC, Canada; 3 Department of Radiation Oncology, BC Cancer—Abbotsford, Abbotsford, BC, Canada; 4 Department of Mathematics and Statistics, University of the Fraser Valley, Abbotsford, BC, Canada; 5 Department of Medical Oncology, BC Cancer—Abbotsford, Abbotsford, BC, Canada; 6 Department of Medicine, Faculty of Medicine, The University of British Columbia, Vancouver, BC, Canada

**Keywords:** cervical cancer, recurrence, survival outcomes

## Abstract

**Background:**

Factors that impact recurrence in stages IB to IV include larger tumor, high-risk histology, older age, and lymphovascular invasion (LVI); however, local studies on risk factors for recurrence in British Columbia and our local recurrence patterns have not been well studied. Furthermore, the efficacy of treatment modalities including surgery and chemoradiation in the different stages of cervical cancer have not been clarified in this population.

**Objectives:**

The purpose of this study is to determine the disease and treatment characteristics of stages IB to IV cervical cancer which are associated with survival differences within British Columbia.

**Methods/Design:**

We performed a retrospective population study. A chart review on cervical cancer patients in British Columbia between 1 January 2010 and 31 December 2017 was done. Demographic data and treatment details were collected. Data were analyzed using multivariate Cox regressions, pairwise comparison using the Log-Rank test, and chi-square tests.

**Results:**

We included 780 patients (stage I: 31.5%, II: 20.0%, III: 34.5%, and IV: 3.3%). LVI and p16 negativity were associated with decreased overall survival (OS), and multivariate analyses show them to be independent risk factors for poorer survival. Surgical resection in stage I was associated with improved survival, but not with stages II–IV. The use of radical radiation therapy (RT), brachytherapy, and concurrent chemotherapy were independently associated with improved survival in stages II–IV. Peri-RT chemotherapy was not associated with survival benefit in adeno/adenosquamous carcinoma. There were 180 recurrences (23.1%), mostly distant metastases (42.8%). There were fewer recurrences after resection of tumors <2 cm compared to tumors 2 cm or larger (6.49% vs 31.3%, p = 0.00011). Only 37.7% of recurrence/metastases were treated with first-line carboplatin/paclitaxel/bevacizumab, but it was associated with better OS compared to other regimens (median OS 40.1 vs 24.8 months, p = 0.03).

**Conclusion:**

A significant portion of patients with localized cervical cancer relapse despite radical therapy, with LVI and p16 negativity associated with poorer survival. Surgical resection may still play a role in stage IB disease, while RT, brachytherapy, and concurrent chemotherapy should be considered first-line therapy in stage II–IV diseases. First-line carboplatin, paclitaxel, and bevacizumab for recurrence shows improved survival.

## Introduction

The lifetime probability of developing cervical cancer is 1 in 168 Canadian women despite decreases in the age-standardized incidence rate (ASIR) of cervical cancer due to screening and human papilloma virus (HPV) vaccination programs.^1^ In British Columbia between 2011 and 2015, 11.8% of cervical cancer cases were diagnosed as Stage IV (metastatic), for which the prognosis is poor.^2^ The CONCORD-2 study reported a 5-year survival rate of 16% in those diagnosed with distant metastases compared to 86% in localized disease.^1^ In the literature, risk factors for recurrence include larger tumor, high-risk histology, older age, and lymphovascular invasion (LVI).^3,4^ Negative P16 expression in HPV-positive cervical cancers is associated with poor survival.^5^ Surgical resection is indicated in early-stage disease (IB or lower), and primary chemoradiation for those stages IB2 to IV.^6^ First-line chemotherapy for recurrence or metastatic cancers includes a combination of cisplatin or carboplatin with paclitaxel,^7^ plus bevacizumab, after the Gynecology oncology group (GOG) 204 study found that the addition of bevacizumab improved the overall survival (OS).^8^ Recently, the anti-programmed cell death-1 (PD-1) inhibitor cemiplimab has been shown to improve OS in recurrent or metastatic cervical cancer after first-line chemotherapy when compared to single-agent chemotherapy.^9^

Local studies on the impact of the histological disease risk factors in cervical cancer recurrence in British Columbia needs to be clarified. Furthermore, survival outcomes associated with treatment modalities including surgery and chemoradiation, by stage, have not been explored in detail within our local population. The purpose of this study is to determine the disease and treatment characteristics of stage IB to IV cervical cancer in British Columbia that are associated with survival differences or recurrence, with specific subgroup analyses for stage IB patients.

## Materials and methods

We conducted a retrospective chart review on patients diagnosed with cervical cancer in the province of British Columbia, Canada, between 1 January 2010 and 31 December 2017. We included all records of consecutive patients available for review through the provincial cancer registry in British Columbia. We excluded patients who were found to have a non-cervical primary, those who had insufficient information on chart review to determine the stage of the patient, or those who were lost to follow-up due to geographic relocation. The ethical approval for this project was obtained from the British Columbia Cancer Agency Research Ethics Board (UBC BCCA REB), H19-03422; written informed consent was not required because of the retrospective nature of this study. Patients were staged according to the 2018 FIGO guidelines.^10^ Demographic data, treatment details, and covariates of prognostic significance were collected using standardized database collection templates. OS was calculated as the time in months from the date of diagnosis to either the last follow-up date, data cut-off date (January 1, 2021), or date of death. Progression-free survival (PFS) was defined as the time in months from the date of diagnosis to either the last follow-up date, data cut-off date, date of cancer recurrence, or death. Cancer-specific survival (CSS) was defined as the time in months from the date of diagnosis to either the last follow-up date, data cut-off date, or date of death caused by cervical cancer.

### Statistical analysis

Data analysis included multivariate Cox regressions, pairwise comparison using the Log-Rank test, chi-square tests, and Fishers exact tests as appropriate. We conducted both univariate and multivariate analyses. Statistical analysis was performed on the *R* programming language (version 3.6.3).^11^

## Results

We initially identified 801 patients: 5 patients were omitted due to endometrial primary, 4 were omitted due to another organ primary (lung, colon, and lymphoma), and 12 were omitted due to insufficient information to stage the patient or loss to follow-up from geographic relocation of the patient, for a total of 780 patients included in the final analyses. The median age was 52 years (range: 25–94 years), with 247 patients (31.6%) diagnosed at stage I, 156 (20.0%) at stage II, 270 (34.5%) at stage III, 104 (13.3%) at stage IV, and 3 (0.5%) with recurrent/unknown disease stage (Table 1). Further clinical and treatment characteristics of the cohort are presented Table 1.

**Table 1.** Clinicodemographic factors and treatment in the cohort.

Characteristic	n (%^a^)
Stage at diagnosis	
I	247 (31.6)
II	156 (20.0)
III	270 (34.5)
IV	104 (13.3)
Recurrent or unknown	3 (0.5)
Age	
<40	149 (19.1)
40+	631 (80.9)
Tumor Size	
<2 cm	108 (13.9)
2 to <4 cm	220 (28.2)
4 cm	416 (53.3)
Missing	36 (4.6)
Smoking	
Non-smoker	382 (49.0)
Current smoker	167 (21.4)
Former smoker	165 (21.2)
Unknown	66 (8.4)
Histology	
Squamous cell carcinoma	569 (72.9)
Adenocarcinoma	191 (24.5)
Other	19 (2.4)
Missing	1 (0.1)
Lymphovascular Invasion	
Absent	240 (30.8)
Present	132 (16.9)
Unknown	408 (52.3)
P16 status	
Negative	21 (2.7)
Positive	208 (26.7)
Unknown	551 (70.6)
Surgery	
No	593 (76)
Yes	187 (4.0)
Surgical treatment	
Primary resection	146 (18.7)
Clearing hysterectomy	41 (5.3)
Lymph nodes	
Negative	74 (9.5)
Positive	25 (3.2)
Unknown	88 (11.3)
Radiation therapy (RT)	
No	140 (17.9)
Yes	640 (82.1)
Type of radiotherapy	
EBRT	194 (24.9)
EBRT + brachytherapy	523 (67.1)
No RT	63 (8.1)
Concurrent Chemotherapy	
No	96 (12.3)
Yes	544 (69.7)
Not applicable	140 (17.9)
Chemotherapy type^b^	
Cisplatin	538 (69.0)
Other	26 (3.3)
Not applicable	229 (29.3)
Weeks of chemotherapy^c^	
5 weeks	408 (52.3)
<5 weeks	143 (18.3)
Not applicable	229 (29.3)
Chemotherapy dose reduction	
No	553 (70.9)
Yes	87 (11.2)
Not applicable	140 (17.9)

RT: radiation therapy; EBRT: external beam radiation therapy.

a Percent of total cohort (n = 780).

b Concurrent chemotherapy.

c Concurrent cisplatin.

**Figure 1.**
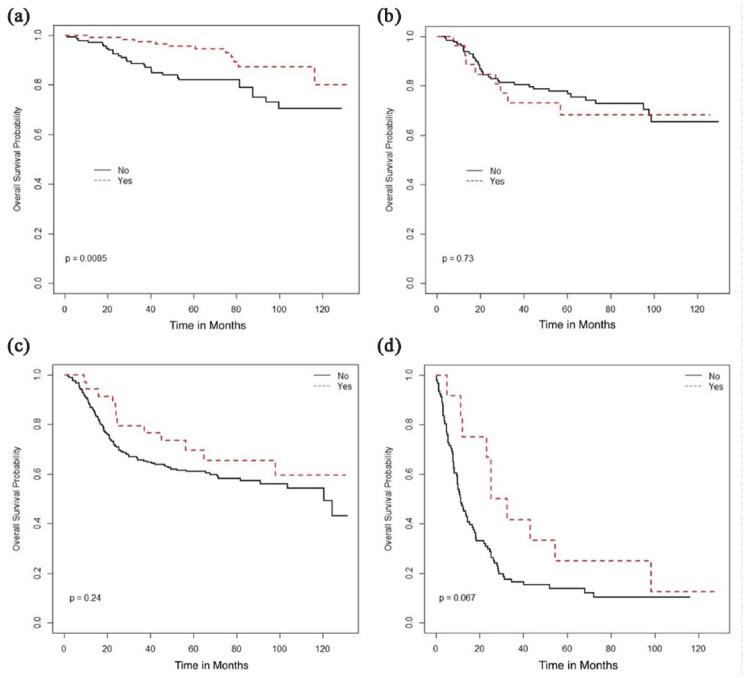
Overall survival (months) based on surgical resection: (a) Stage I, (b) Stage II, (c) Stage III, and (d) Stage IV.

**Figure 2.**
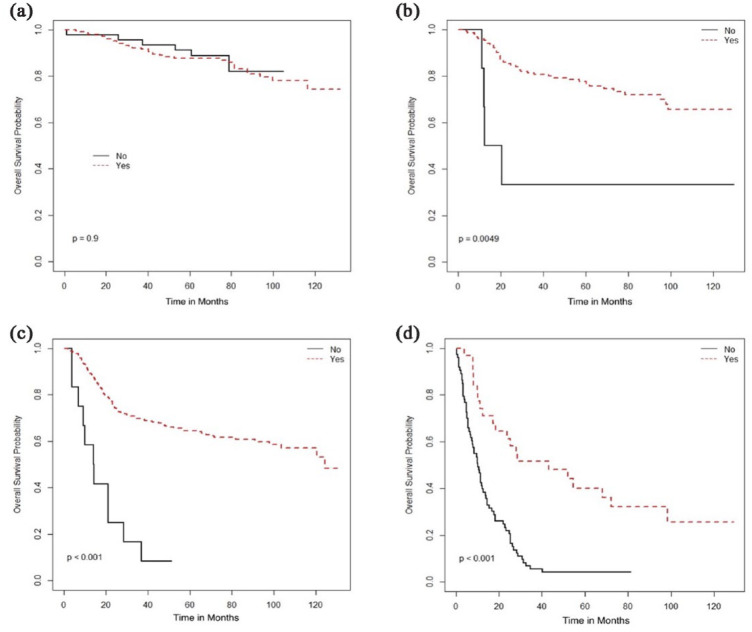
Overall survival (months) based on radical radiotherapy: (a) Stage I, (b) Stage II, (c) Stage III, and (d) Stage IV.

**Figure 3.**
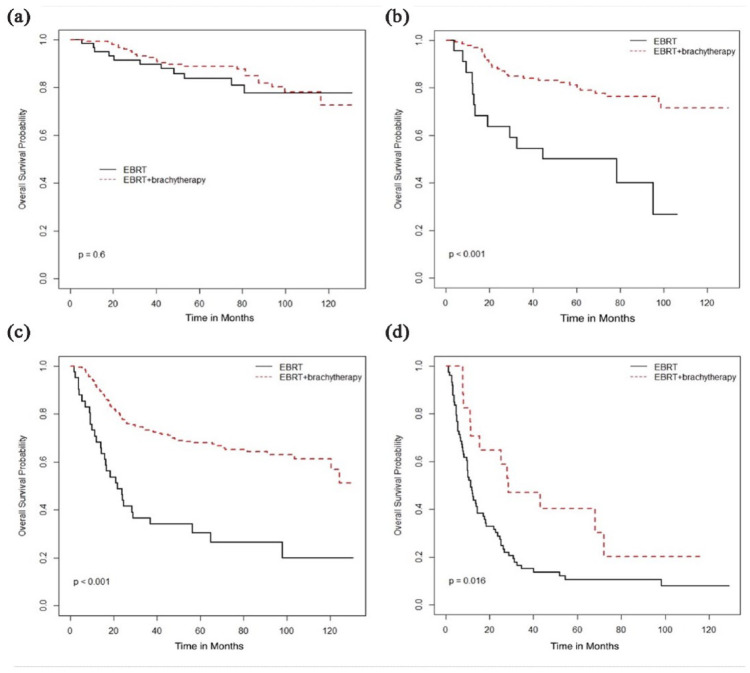
Overall survival (months) based on type of radiotherapy (EBRT vs EBRT + brachytherapy): (a) Stage I, (b) Stage II, (c) Stage III, and (d) Stage IV.

**Figure 4.**
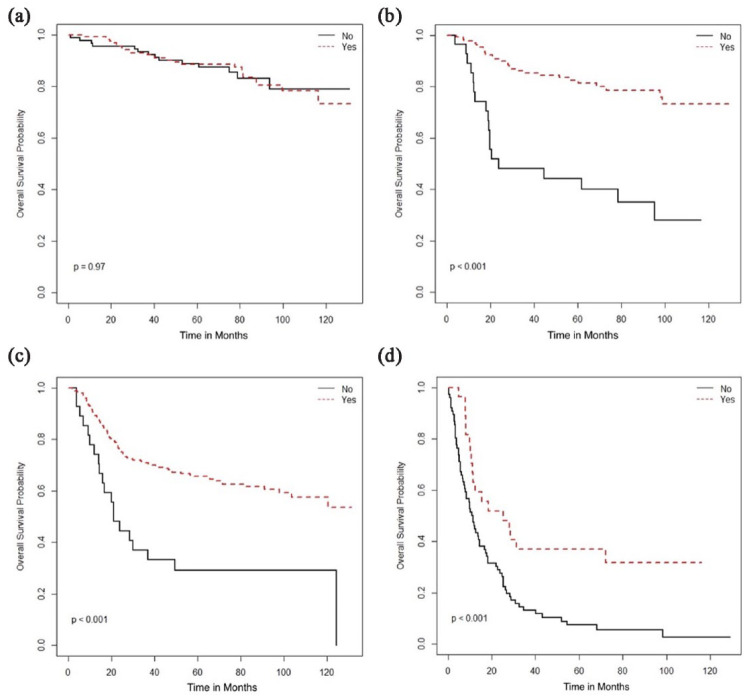
Overall survival (months) based on use of concurrent chemotherapy: (a) Stage I, (b) Stage II, (c) Stage III, and (d) Stage IV.

Surgical resection was associated with better OS in stage I patients but not in any other stage (Figure 1, Table 2). Other treatment associated with stage-based survival benefit was the use of radical radiation therapy (RT), addition of brachytherapy with external beam radiation therapy (EBRT), and concurrent chemotherapy, which was associated with improved OS in stage II–IV diseases (Figures 2–4) but not in stage I patients.

**Table 2.** Median overall survival (months) based on disease and treatment factors, stratified by stage at diagnosis.

Characteristic		Median overall survival (months)							
		P		P		P		P	
		Stage I	Value	Stage II	Value	Stage III	Value	Stage IV	Value
Lymphovascular invasion	Yes	NR		NR		104		12.2	
	No	NR	0.005	NR	0.05	NR	0.04	23.7	0.21
	Unknown	NR		NR		121		12.0	
P16 status	Positive	NR		NR		71.6		11.70	
	Negative	NR	0.74	24.2	0.01	16	<0.001	8.23	0.19
	Unknown	NR		NR		124.4		15.36	
Surgical resection	Yes	NR	0.009	NR	0.73	NR	0.24	28.9	0.067
	No	NR		NR		121		11.4	
Radical radiotherapy	Yes	NR	0.90	NR	0.005	124.4	<0.001	43.2	<0.001
	No	NR		16.5		14.3		10.0	
Type of RT	EBRT+								
	Brachytherapy	NR	0.60	NR	<0.001	NR	<0.001	28.5	0.02
	EBRT alone	NR		61.5		22		11.5	
Concurrent chemotherapy	Yes	NR	0.97	NR	<0.001	NR	<0.001	25.3	–0.001
	No	NR		23.8		21.2		11.2	
Concurrent chemotherapy type	Weekly Cisplatin	NR	0.32	NR	0.65	NR	0.15	21.9	0.19
	Other	81.5		NR		39.8		14.5	
Weeks of concurrent cisplatin	5 weeks	NR	0.16	NR	0.003	NR	0.007	28.1	0.78
	<5 weeks	NR		NR		65		15.5	

NR: not reached; RT: radiation therapy; EBRT: external beam radiation therapy.

a NR (not reached) = the 50% survival point of that group has not been reached.

**Figure 5.**
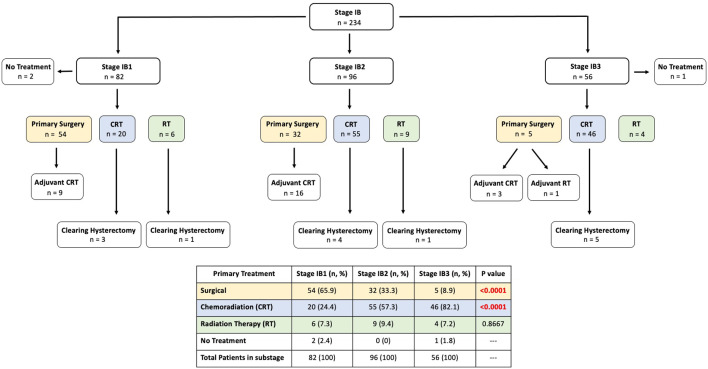
Flowchart of treatment of stage IB patients with comparison of primary treatment (surgical, chemoradiation, and radiation) in stage IB1 versus IB2 versus IB3.

We investigated if the improved survival outcomes in stage I patients was attributable to a specific subgroup but found no significant differences in OS or PFS between stage IB1, IB2, and IB3 patients who received primary surgical treatment. A treatment flowchart for the 234 stage IB patients is presented in Figure 5, consisting of 82/234 patients (35.0%) that were stage IB1, 96/234 (41.0%) stage IB2, and 56/234 (24.0%) stage IB3. There was no significant difference in histologies between the substages (p = 0.055). There was however, significant differences in proportions of women receiving either primary surgical treatment and primary chemoradiation therapy (CRT) between the stage IB1, IB2, and IB3 (p < 0.0001). Stage IB1 patients were mostly managed with surgery (54/82, 65.9%) with only 20/82 (24.4%) receiving primary CRT (Figure 5). In comparison, over half of stage IB2 patients were treated with primary CRT (55/96, 53.1%) and in stage IB3, the majority (46/56, 82.1%) received primary CRT with very few receiving primary surgery (5/56, 8.9%). When considering adjuvant treatment, there was significantly more RT, either as primary/adjuvant CRT/RT in stage IB3 compared to stage IB2 and stage IB1 (stage IB3: 54/56 = 96%; stage IB2: 80/96 = 83.3%; stage IB1: 35/82 = 42.6%, p < 0.00001).

Multivariate analyses of the entire cohort for OS, PFS, and CSS (Table 3) showed a lower CSS for non-squamous/adenocarcinoma histologies with a hazard ratio (HR) of 0.18 (p = 0.032), but no significant differences in OS or PFS between any of the different histologies. LVI, which was present in 132 women (16.9%), absent in 240 (30.8%), and unknown in 408 (52.3%) was associated with a worse PFS and OS. In our cohort, 208 had p16-positive tumors (26.7%), 21 were p16 negative (2.7%) and 551 were unknown (70.6%). Absent or unknown p16 was associated with poorer OS and PFS. Surgery was associated with better OS, PFS, and CSS, while use of RT was associated with better PFS only. Brachytherapy in addition to EBRT was associated with better OS, PFS, and CSS. Concurrent chemotherapy was associated with better OS and CSS. Less than 5 weeks of concurrent chemotherapy was associated with worse OS and PFS than 5 weeks of chemotherapy, while dose reduction did not have a significant impact on survival.

**Table 3.** Multivariate analysis of demographic and disease factors in the cohort.

Characteristic	OS		PFS		CSS	
	HR (95% CI)	p value	HR (95% CI)	p value	HR (95% CI)	p value
Stage at diagnosis						
I	Reference	–	Reference	–	Reference	–
II	1.11 (0.62–1.98)	0.722	1.02 (0.58–1.82)	0.935	1.73 (0.69–3.41)	0.299
III	2.41 (1.51–3.85)	0.0002	2.34 (1.46–3.73)	<0.0004	2.17 (1.12–4.22)	0.022
IV	3.30 (1.53–7.12)	0.002	4.85 (2.37–9.92)	<0.0001	1.69 (0.67–4.25)	0.262
Age						
<40	Reference	–	Reference	–	Reference	–
40+	1.10 (0.72–1.67)	0.67	0.93 (0.61–1.42)	0.743	0.64 (0.39–1.04)	0.074
Tumor Size						
<2 cm	Reference	–	Reference	–	Reference	–
2+ cm	2.61 (0.94–7.26)	0.065	3.04 (1.09–8.46)	0.033	1.75 (0.21–14.76)	0.609
Smoking						
Non-smoker	Reference	–	Reference	–	Reference	–
Current smoker	1.04 (0.69–1.57)	0.841	0.96 (0.63–1.45)	0.836	1.23 (0.77–1.98)	0.392
Former smoker	1.04 (0.68–1.59)	0.861	0.93 (0.60–1.44)	0.741	1.64 (0.94–2.86)	0.079
Unknown	0.87 (0.45–1.69)	0.674	0.87 (0.44–1.70)	0.676	1.08 (0.50–2.31)	0.851
Histology						
SCC	Reference	–	Reference	–	Reference	–
Adenocarcinoma	0.91 (0.58–1.44)	0.69	1.05 (0.66–1.66)	0.837	0.75 (0.43–1.33)	0.323
Other	0.34(0.08–1.41)	0.137	0.41 (0.10–1.69)	0.215	0.18 (0.04–0.87)	0.032
Lymphovascular invasion						
Absent	Reference	–	Reference	–	Reference	–
Present	1.81 (1.00–3.28)	0.05	1.93 (1.08–3.47)	0.027	1.97 (0.82–4.71)	0.128
Unknown	2.03 (1.31–3.16)	0.002	1.93 (1.24–3.00)	0.003	1.18 (0.66–2.13)	0.579
P16 status						
Negative	Reference	–	Reference	–	Reference	–
Positive	0.25 (0.12–0.54)	0.0004	0.25 (0.12–0.54)	0.0003	0.64 (0.27–1.53)	0.313
Unknown	0.15 (0.07–0.32)	<0.0001	0.14 (0.06–0.29)	<0.0001	0.36 (0.15–0.90)	0.03
Surgery						
No	Reference	–	Reference	–	Reference	–
Yes	0.46 (0.24–0.86)	0.016	0.38 (0.20–0.71)	0.002	0.35 (0.15–0.82)	0.016
Radiation therapy (RT)						
No	Reference	–	Reference	–	Reference	–
Yes	0.45(0.16–1.27)	0.131	0.35 (0.13–0.94)	0.037	1.61 (0.38–6.94)	0.52
Type of radiotherapy						
EBRT	Reference	–	Reference	–	Reference	–
EBRT + brachy	0.42 (0.23–0.76)	0.005	0.41 (0.24–0.68)	0.0006	0.37 (0.20–0.70)	0.002
Concurrent chemotherapy						
No	Reference	–	Reference	–	Reference	–
Yes	0.013 (0.002–0.11)	<0.0001	0.31 (0.04–2.34)	0.257	0.02 (0.002–0.19)	0.0006
Chemotherapy type^a^						
Cisplatin	Reference	–	Reference	–	Reference	–
Other	0.58 (0.20–1.71)	0.323	0.54 (0.18–1.60)	0.265	0.65 (0.15–2.80)	0.561
Weeks of chemotherapy^b^						
5 weeks	Reference	–	Reference	–	Reference	–
<5 weeks	1.75 (1.25–2.45)	0.001	1.67 (1.19–2.35)	0.003	1.47 (0.96–2.25)	0.074
Chemotherapy dose reduction						
No	Reference	–	Reference	–	Reference	–
Yes	1.25 (0.82–1.92)	0.3	1.14 (0.74–1.75)	0.545	1.44 (0.80–2.60)	0.226

OS: overall survival; PFS: progression-free survival; CSS: cancer-specific survival; HR: hazard ratio; CI: confidence interval; SCC: squamous cell carcinoma; RT: radiation therapy; EBRT: external beam radiation therapy.

a Concurrent chemotherapy.

b Concurrent cisplatin.

**Figure 6.**
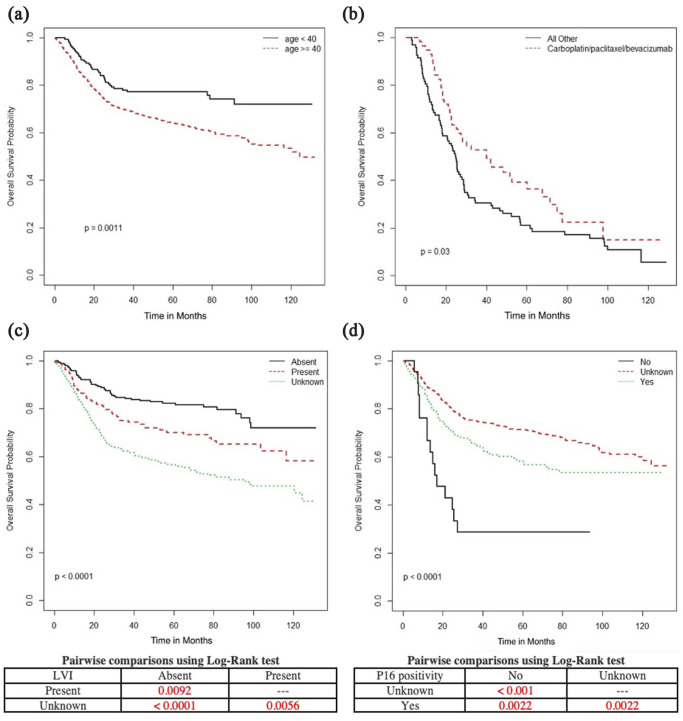
Overall survival (months) in the entire cohort according to (a) Age (<40 vs age 40 or older), (b) first-line systemic therapy in recurrence or metastatic cases (carboplatin, paclitaxel, and bevacizumab vs all other therapies), (c) lymphovascular invasion (absent, present, or unknown), and (d) P16 positivity (yes, no, unknown).

**Figure 7.**
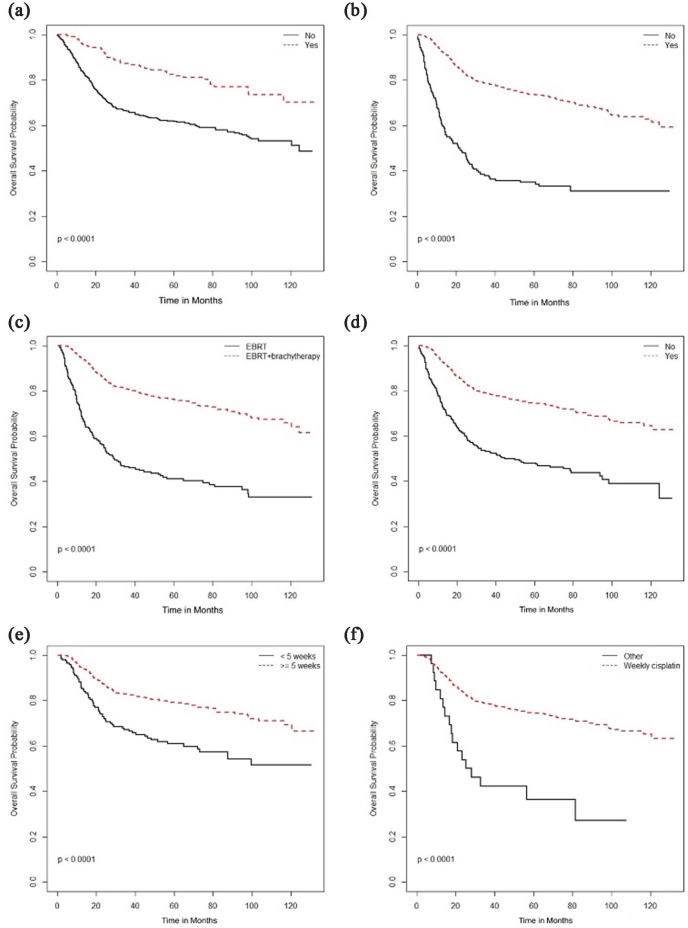
Overall survival (months) and association with (a) surgical resection, (b) radical radiotherapy (RT), (c) type of RT, (d) use of concurrent chemotherapy, (e) weeks of concurrent chemotherapy, and (f) type of concurrent chemotherapy.

**Figure 8.**
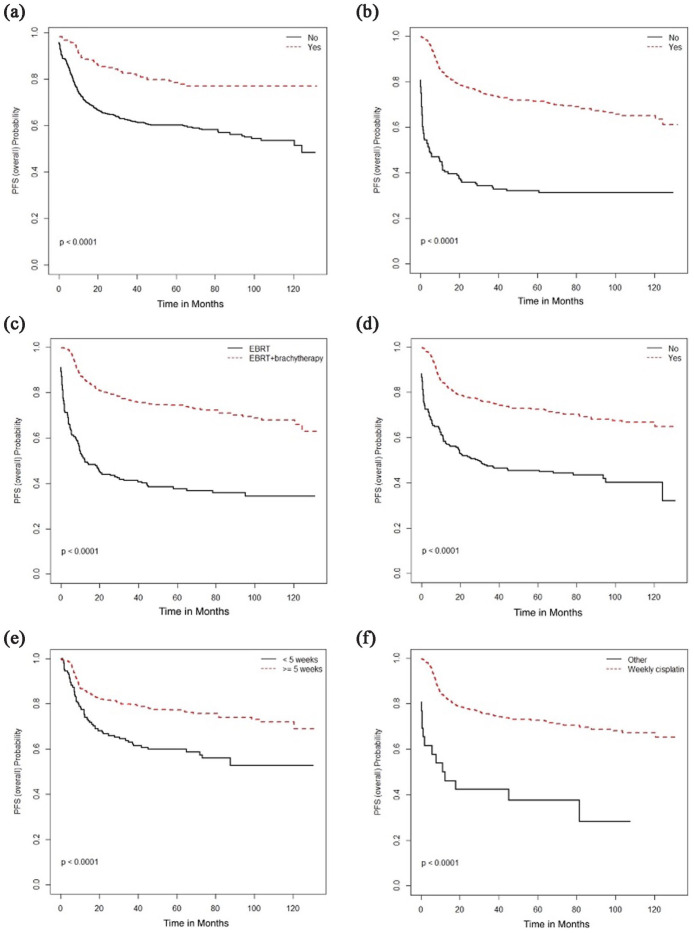
Progression-free survival (months) and association with (a) surgical resection; (b) radical radiotherapy (RT), (c) type of RT, (d) use of concurrent chemotherapy, (e) weeks of concurrent chemotherapy, and (f) type of concurrent chemotherapy.

**Figure 9.**
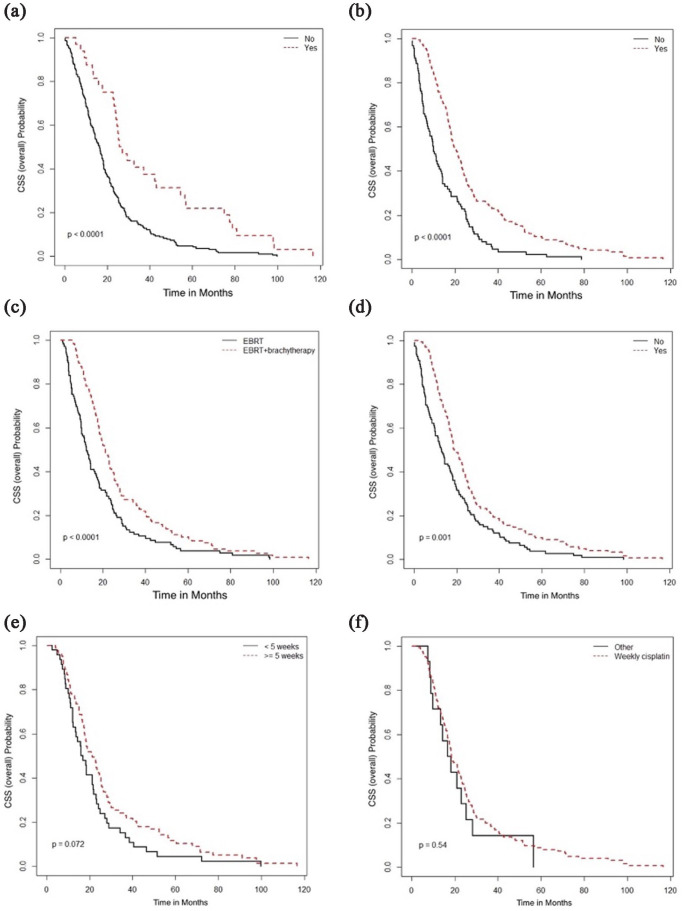
Cancer-specific survival (months) and association with (a) surgical resection, (b) radical radiotherapy (RT), (c) type of RT, (d) use of concurrent chemotherapy, (e) weeks of concurrent chemotherapy, and (f) type of concurrent chemotherapy.

**Figure 10.**
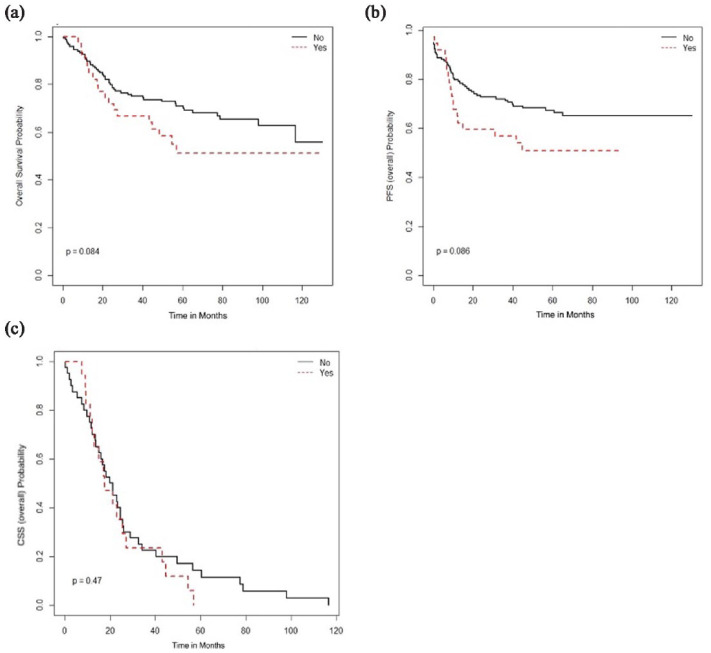
No survival benefit of peri-RT chemo in adenocarcinoma or adenosquamous carcinoma in the study population: (a) overall survival, (b) progression-free survival, and (c) cancer-specific survival.

On univariate analyses of the entire cohort (Figure 6(a), (c) and (d)), decreased OS was associated with age 40+ (vs under 40) (median OS age >40 was 124 months, versus not reached (NR) in those under 40, p = 0.0011), positive LVI (median OS with LVI NR vs 95.1 months when unknown vs NR when absent, p < 0.0001) and p16 negativity (median OS in p16 negative was 17.2 months vs NR in P16 positive or unknown). Consistent with our multivariate analyses, longer OS and PFS was associated surgical resection, radical RT, brachytherapy, concurrent cisplatin, and completion of 5 weeks of chemotherapy (Figures 7 and 8). Surgical resection, radical RT, and brachytherapy were associated with longer CSS (Figure 9). Peri-RT chemotherapy was not associated with survival benefit in adenocarcinoma or adenosquamous carcinoma (Figure 10).

Recurrence after radical therapy was seen in 180 patients (23.1%), 42.8% with distant metastases. The incidence of recurrence after surgical resection was 4.1% if the tumor was <2 cm (n = 77) and 24.7% if the initial tumor was ⩾2 cm (n = 110; p = 0.0002); however, there was no significant difference in recurrence rates at 3 and 4 cm; therefore, in our cohort, stage 2 cm (stage IB1), may be the latest stage where primary surgery has a significant impact on survival. Having regional lymph node involvement at diagnosis did not have a difference in recurrence, with 25% (5/25) recurrence in those with regional lymph nodal involvement at diagnosis and 22.6% (37/162) in those with no or unknown nodal involvement (p = 0.75). Fifty-seven of the 151 women (37.7%) who received systemic therapy for recurrence/metastases were treated with first-line carboplatin, paclitaxel, and bevacizumab. This combination therapy was associated with better OS compared to other regimens (median OS 40.1 vs 24.8 months, p = 0.03, Figure 6(b)). Sixty of 151 women (39.7%) treated for recurrence or metastases went on to second-line systemic therapy, and 25/151 (16.6%) went to receive third-line systemic therapy.

## Discussion

Our study presents a large comprehensive analysis of local treatment and recurrence patterns for stage IB to IV cervical cancer in British Columbia. We found that surgical resection improves OS and likely plays an important role in the management of stage I cervical cancer. A similar study with 1936 stage I cervical cancer patients in China also found increased recurrences in patients who received only chemotherapy compared to those who received surgical resection.^12^ Furthermore, radical hysterectomy improved survival in stage IB2 patients, compared to CRT alone in a review of seven studies.^13^ In our cohort, tumor size < 2 cm was statistically associated with fewer recurrences (6.49% tumors < 2 cm had recurrences vs 31.3%, p = 0.00011). This may be due to decreased parametrial involvement, as tumors < 2 cm had much lower parametrial involvement in a study of 461 stage IB patients^14^ and have also shown a lower risk of being pathologically upstaged after surgical resection.^15^ A study with stage IB1 patients showed that tumors larger than 2.7 cm were associated with increased risk factors like LVI or deep stromal invasion,^16^ suggesting that increasing tumor size corresponds with and correlates with other poor histologic indices. In our cohort, earlier substages like stage IB1 receive primary surgical treatment at a higher proportion (65.9%) compared to stage IB2 or stage IB3 (33.3% and 8.9%, respectively). The opposite trend is seen in CRT, where majority of stage IB3 patients receive CRT (82.1%). Stage IB1 to IB3 patients who received primary surgery all had similar survival outcomes regardless of substage, which could reflect ideal patient selection. Our study adds to the body of literature to show that tumors larger than 2 cm may not be best addressed with surgical resection and could be considered for primary CRT.

To our knowledge, this is the largest Canadian cohort to demonstrate and confirm the survival benefit of chemoradiation in stage II to IV cervical cancer. Brachytherapy was associated with improved survival in our cohort and has been reported in the literature to decrease recurrence, independent of tumor stage.^17^ The benefit of brachytherapy in stage II to IV patients is evident even if the timing of brachytherapy does not occur within the standard 8 weeks of therapy.^18^ Our study also confirms that LVI, older age, and p16 negativity remain important risk factors for poor prognosis. Older patients were less likely to be treated with the standard of care, even though completion of standard treatment is known to improve OS at all ages.^19^ Other risk factors for locoregional recurrence in locally advanced cervical cancer include non-squamous histology or positive lymph nodes.^20^ A large study with more than 80,000 patients found adenocarcinoma histology was associated with worse survival compared to squamous carcinomas.^21^ We did not find survival differences between squamous cell carcinomas and adenocarcinomas, which could highlight the greater importance of other risk factors, as a large study of 3298 stage IB/IIA patients reported that deep stromal invasion was associated with higher recurrence and worse OS, with no correlation to tumor histology.^22^ In our cohort, absent p16 was associated with poorer survival. In previous studies, p16 positivity is associated with higher risk of progression in precancerous lesions,^23^ but in invasive cancer, p16 negativity has been associated with advanced stage, older age, and poorer prognosis, potentially from poor tumor differentiation.^15^ There is conflicting evidence whether absent p16 is a risk factor independent of other risk factors.^24^ Our data show that p16 negativity is associated with poorer survival in both univariate and multivariate analyses, suggesting that p16 status may be a molecular prognostic factor independent of other clinical factors.

Our study results are consistent with the recent findings from the randomized phase 3 OUTBACK trial that reported no survival benefit from adjuvant chemotherapy after cisplatin-based chemoradiation.^25^ Adjuvant chemotherapy has been shown to improve disease-free survival and reduce distant metastases in some studies, but not all.^26^ The poor response of adeno/adenosquamous carcinomas to chemoradiation is well reported in multiple studies.^27,28^ In our province, peri-radiation chemotherapy was only offered to patients with adenocarcinoma or adenosquamous histology, based on a previous study that showed that there may be survival advantage to additional chemotherapy after radiation.^26^ Our subgroup of adenocarcinoma or adenosquamous histology did not seem to benefit from additional chemotherapy, although our study may not be powered for subgroup analysis. With the level-1 evidence now demonstrating no benefit of adjuvant chemotherapy to primary chemoradiation therapy in invasive cervical cancer, our study adds to the real-world evidence that even in adenocarcinoma or adenosquamous histology, the benefit of adjuvant chemotherapy is not demonstrated.

In our cohort, patients with stage-IV cancer have limited survival and although carboplatin, paclitaxel, and bevacizumab are the current standard of care for the first-line treatment of advanced or recurrent cervical cancer,8,29 only 37.7% of women were treated with first-line therapy. We also saw 39.7% of women go on to second-line chemotherapy, of which no survival benefits have been reported.^30^ This shows the importance of using the most effective treatment upfront, as most patients will not proceed to further therapy after their first option. Early results from a randomized control trial (RCT) showed improved survival with cemiplimab compared to single agent chemotherapy with pemetrexed, vinorelbine, gemcitabine, irinotecan, or topotecan.^9^ The incorporation of immunotherapy into the palliative treatment strategy is likely to change the landscape of systemic therapy for recurrent and metastatic cervical cancer in the future.

### Limitations

Our study is a large multi-center population cohort, which reduces selection bias encountered in single-site cohorts. The retrospective nature of our cohort has inherent confounding biases from the methodology however, our large sample size and the use of multivariate analyses attempt to address those aspects. Our analysis on surgical resection does not factor in the timing of surgical resection, as both primary resection and adjuvant clearing hysterectomies are considered together.

## Conclusion

Our cohort of cervical cancer patients demonstrates the survival benefit of chemoradiation with brachytherapy in stage II to IV cervical cancer, where chemoradiation is the mainstay of radical treatment. We found a survival benefit with surgery in stage I, where it is utilized proportionally more in stage IB1 compared to stage IB2 or IB3, and tumors <2 cm are associated with less recurrence. Histological factors including LVI and p16 negativity are associated with worse survival outcomes. The recurrence rate was in 39.7% of our cohort where current first-line systemic therapy of carboplatin, paclitaxel, and bevacizumab demonstrated improved OS compared to all other single or multi-agent systemic therapies and should be the preferred treatment regimen.

## Declarations

**Ethics approval and consent to participate:** The ethical approval for this project was obtained from the British Columbia Cancer Agency Research Ethics Board (UBC BCCA REB), H19-03422, written informed consent was not required because of the retrospective nature of this study.

**Consent for publication:** Not applicable.

**Availability of data and materials:** The primary data are available from the authors upon request.
